# Lymph Node Dissection Is a Risk Factor for Short-Term Cough after Pulmonary Resection

**DOI:** 10.3390/curroncol29010027

**Published:** 2022-01-10

**Authors:** Xiaoli Wu, Hanyang Xing, Ping Chen, Jihua Ma, Xintian Wang, Chengyi Mao, Xiaoying Zhao, Fuqiang Dai

**Affiliations:** 1 Department of Thoracic Surgery, Daping Hospital (Army Medical Center of PLA), Third Military Medical University, Chongqing 400042, China; ffx5201212@163.com (X.W.); lzw712712@sina.com (P.C.); m122909612@163.com (J.M.); wxt000926@163.com (X.W.); 2 Department of Gastroenterology, Daping Hospital (Army Medical Center of PLA), Third Military Medical University, Chongqing 400042, China; xinghanyang00@163.com; 3 Department of Pathology, Daping Hospital (Army Medical Center of PLA), Third Military Medical University, Chongqing 400042, China; maochengyi3636@163.com

**Keywords:** postoperative cough, pulmonary resection, video-assisted thoracoscopic surgery (VATS), risk factor analysis

## Abstract

Cough is a common complication after pulmonary resection. However, the factors associated with cough that develop after pulmonary resection are still controversial. In this study, we used the Simplified Cough Score (SCS) and the Leicester Cough Questionnaire (LCQ) score to investigate potential risk factors for postoperative cough. Between January 2017 and June 2021, we collected the clinical data of 517 patients, the SCS at three days after surgery and the LCQ at two weeks and six weeks after surgery. Then, univariate and multivariate analyses were used to identify the independent risk factors for postoperative cough. The clinical baseline data of the cough group and the non-cough group were similar. However, the cough group had longer operation time and more blood loss. The patients who underwent lobectomy were more likely to develop postoperative cough than the patients who underwent segmentectomy and wedge resection, while the patients who underwent systematic lymph node dissection were more likely to develop postoperative cough than the patients who underwent lymph node sampling and those who did not undergo lymph node resection. When the same lymph node management method was applied, there was no difference in the LCQ scores between the patients who underwent wedge resection, lobectomy and segmentectomy. The lymph node resection method was an independent risk factor for postoperative cough (*p* < 0.001). Conclusions: Lymph node resection is an independent risk factor for short-term cough after pulmonary resection with video-assisted thoracoscopic surgery, and damage to the vagus nerve and its branches (particularly the pulmonary branches) is a possible cause of short-term cough. The mechanism of postoperative cough remains to be further studied.

## 1. Introduction

With the development of thoracoscopic surgery techniques, most pulmonary resection surgeries [1,2], even including pulmonary sleeve resection [3], can be achieved through minimally invasive thoracoscopic surgery. Minimally invasive thoracoscopic surgery has the advantages of minimal trauma, a good aesthetic effect and enhanced recovery. However, a considerable number of patients still develop cough symptoms after pulmonary resection with minimally invasive thoracoscopic surgery [4,5], which may affect postoperative recovery and quality of life [6].

Cough is a protective reflex of the respiratory tracts [7]. Many organs of the human body are involved in cough, such as sensory neurons and afferent nerves (vagus nerve, glossopharyngeal nerve, etc.), efferent nerves (spinal nerve, phrenic nerve, etc.) and effector organs (respiratory muscle, diaphragm and glottis). A pathological change in one of these elements may cause cough [8]. Pulmonary resection may be involved in damaging components in the larynx and thorax. Previous studies have shown that postoperative cough may be related to lobectomy, subcarinal lymph node dissection, postoperative acid reflux, duration of anesthesia [9], the area submitted to pulmonary resection, history of chronic obstructive pulmonary disease (COPD) [10], and anesthesia time [11]. However, the factors associated with cough symptoms after pulmonary resection are still controversial.

In our study, we investigated the relationship between postoperative cough after pulmonary resection and patient clinical characteristics and perioperative parameters based on the Mandarin Chinese version of the Leicester Cough Questionnaire (LCQ-MC) and the Simplified Cough Score (SCS). In addition, the potential risk factors for postoperative cough were explored with the aim of reducing the incidence of cough symptoms in patients after pulmonary resection.

## 2. Materials and Methods

A retrospectively observational study was conducted in patients who underwent pulmonary resection by the same surgical group (Daping Hospital, Third Military Medical University). This study was supported by the ethics committee of the hospital (approval number: 2021–235). All patients had signed a written informed consent form before treatment. This study was conducted in accordance with the Declaration of Helsinki (2013 revision).

### 2.1. Patient Selection

A total of 517 patients with lung tumors who were admitted to the department of thoracic surgery of the Third Affiliated Hospital (Daping Hospital) of the Third Military Medical University between January 2017 and June 2021 were selected. They all met the following inclusion criteria: (I) males or females aged 20–80 years; (II) single-port or two-port video-assisted thoracoscopic surgery (VATS) lobectomy, segmentectomy, or pulmonary wedge resection; (III) no pulmonary infection or inflammation, including chronic obstructive pulmonary disease (COPD) in medical history; (IV) no cough symptoms within two weeks before surgery (preoperative cough may be caused by pulmonary inflammation, a large tumor or tumor in the large bronchi) and the cough would have needed to begin on the first day after the operation and was defined and assessed by our research group based on previous studies [12]; (V) no distant metastasis.; (VI) pathological diagnosis; and (VII) informed consent form signed before surgery. Patients who met the following criteria were excluded: (I) poor cardiopulmonary function and intolerance of surgery due to cardiopulmonary or other organ dysfunction; (II) conversion to thoracotomy from VATS; (III) severe postoperative complications, including severe infections, pulmonary embolism, chylothorax, vocal cord paralysis and hoarse voice; and (IV) refusal to undergo follow-up. Data regarding age, sex, forced expiratory volume in 1 s (FEV1), predicted percentage of the forced vital capacity (FEV1%), body mass index (BMI), Charlson comorbidity index (CCI), operation time, postoperative pathology, and drinking and smoking status were collected and analyzed.

### 2.2. Surgical Techniques

The patients underwent general anesthesia and double-lumen endotracheal intubation in the contralateral decubitus position before single-port or two-port thoracoscopic surgery. An operating port with a size of 3–5 cm was placed at the fourth or fifth intercostal space in the anterior axillary line in all patients. The camera was placed in an incision at the seventh intercostal space in the anterior axillary line, which refers to the two-port VATS, or at the posterior end of the operating incision, which refers to the single-port VATS. Lobectomy or sublobular resection, including wedge resection and segmentectomy, was selected as the surgical method according to preoperative image data and intra-operative rapid frozen section examination. Lymph nodes were grouped according to the eighth edition lung cancer stage classification [13] and were resected or sampled according to the Chinese Guidelines for the Diagnosis and Treatment of Primary Lung Cancer (2018) [14]. In short: for benign disease, neither lymph node resection nor sampling was performed; for carcinoma in situ or micro invasive carcinoma, local lymph node sampling was performed after pulmonary segmentectomy; and for invasive carcinoma, systematic hilar and mediastinal lymph node dissection was performed.

### 2.3. Postoperative Management

All patients were sent to the thoracic surgery unit after surgery (after the operation, patients were required to stay for a short time in the recovery room and were then transferred to the ward). Professional nurses recorded the postoperative parameters of the patients, including symptoms, vital signs, draining materials, 24-h drainage volume, urine volume, etc. After surgery, the patients were encouraged to cough and expectorate to promote drainage and pulmonary re-expansion and were instructed to undertake early activities after surgery. Chest radiographs and routine blood tests were performed in the first day after surgery. Patients whose 24-h chest drainage volume was less than 200 mL, and who had no pneumothorax or residual space on a chest radiograph as well as no air leakage from the chest tube underwent chest tube removal.

### 2.4. Evaluation Methods and Grouping Criteria for Cough

The Mandarin Chinese Version of the LCQ [15] was administered to all enrolled patients at 2 weeks and 4 weeks postoperatively via the outpatient department under the guidance of two trained members of the thoracic medical staff. The LCQ scale is highly effective for evaluating chronic and acute cough [16,17,18] and is easy to complete and can be self-administered in less than 5 min. A total score and three domain scores were calculated with higher scores indicating better health [19].

Postoperative cough was evaluated using the Simplified Cough Score (SCS) on the third day after surgery [20]. The SCS includes the daytime and nighttime SCSs. Each item is scored from 0 to 3 points according to severity. Patients with an SCS of 0 or 1 were assigned to the non-cough group, and patients with an SCS of 2 or 3 were assigned to the cough group.

### 2.5. Statistical Analysis

Firstly, we compared the clinical data and treatment outcomes between the cough and non-cough groups, and conducted multivariate analyses to explore the independent risk factors for postoperative cough. Then, the two groups were compared in the lobectomy subgroup and the non-small cell lung cancer (NSCLC), respectively. Data analysis was performed using SPSS 18.0 software (Statistical Package for the Social Sciences, Chicago, IL, USA). Continuous data are presented as mean ± standard deviation (SD), and categorical data are presented as frequency and percentage (%). Univariate analysis (the chi-square test, t-test) was used to evaluate the possible risk factors for postoperative cough, and a multivariate analysis (logistic regression test, variables whose *p*-value less than 0.1 were included) was performed to determine the independent risk factors. In the Chi-square test, if all theoretical frequencies were T ≥ 5 and total samples *n* ≥ 40, then Pearson Chi-square test was used. If 1 < T < 5 and *n* ≥ 40, then continuity correction Chi-square test was used. If T < 1 or *n* < 40, Fisher’s accurate test was used. When conducting the analysis of continuous data, if the sample conformed to normal distribution and had homogeneity of variance, the Student’s T-test was used. If it conformed to normal distribution but did not have homogeneity of variance, Welch’s t-test was used. If it didn’t conform to normal distribution, Mann–Whitney U test, a kind of rank sum test, was used. *p* < 0.05 was considered significant.

## 3. Results

### 3.1. Patients’ Clinical and Pathological Data and Univariate Analysis of Postoperative Cough

Of a total of 517 enrolled cases, 310 (59.96%) were assigned to the non-cough group and 207 (40.04%) were assigned to the cough group. Baseline characteristics of all cases are presented in Table 1. The two groups were similar in terms of sex, age, smoking status, drinking status, BMI, FEV1, FEV1%, CCI and tumor location. The majority of the patients were male. The tumor size was larger in the cough group than in the non-cough group, but the difference was not significant (2.44 ± 3.79 cm vs. 2.03 ± 0.91 cm, *p* = 0.067).

### 3.2. Treatment Results

Different incisions had no significant effect on postoperative cough. The patients in the non-cough group had a shorter operation time (122.03 ± 53.55 min vs. 146.11 ± 46.72 min; *p* < 0.001) (Table 2), less blood loss (113.85 ± 71.47 mL vs. 142.46 ± 106.01 mL; *p* < 0.001), less drainage time (3.87 ± 2.33 days vs. 4.34 ± 2.96 days; *p* = 0.028) and a shorter hospital stay (7.58 ± 3.84 days vs. 8.32 ± 3.81 days; *p* = 0.034). The incidence of postoperative cough in patients with benign disease was lower than that in patients with malignant tumors (22.4% vs. 52.1%, χ^2^ = 45.929, *p* < 0.001). In addition, the incidence of cough was higher in the following instances: after pulmonary segmentectomy, higher than after wedge resection (37.8% vs. 16.0%, χ^2^ = 11.629, *p* = 0.001); after lobectomy, higher than after segmentectomy (47.8% vs. 37.8%, χ^2^ = 3.906, *p* = 0.048); and after lobectomy, higher than after wedge resection (47.8% vs. 16.0%, χ^2^ = 26.433, *p* <0.001). Regarding different lymph node management methods, the incidence of cough was higher in the patients who underwent systematic lymph node dissection than in those who underwent lymph node sampling (67.9% vs. 41.3%, χ^2^ = 21.254, *p* < 0.001) and in those who did not undergo lymph node resection (67.9% vs. 14.4%, χ^2^ = 118.324, *p* < 0.001). The incidence of cough was higher in the patients who underwent lymph node sampling than in those who did not undergo lymph node resection (41.3% vs. 14.4%, χ^2^ = 30.347, *p* < 0.001).

**Figure 1:**
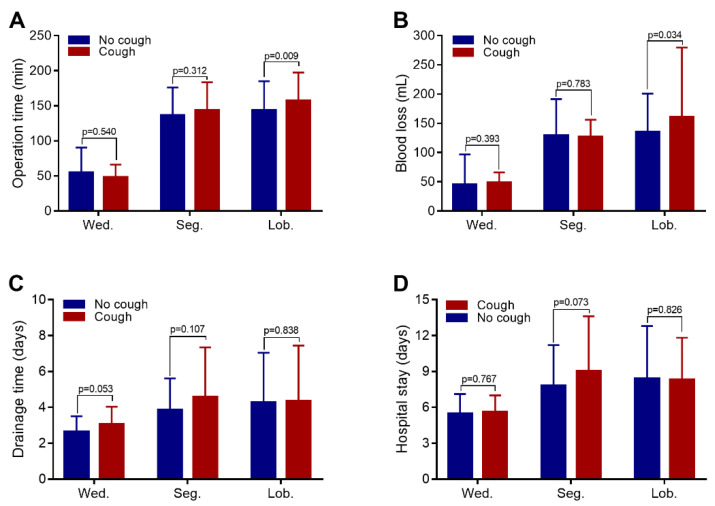
Comparison of surgical parameters between the cough and non-cough groups with different types of pulmonary resection. (**A**) There were no significant differences in operation time between the patients who underwent wedge resection (Wed.) and the patients who underwent segmentectomy (Seg.). However, among those who underwent lobectomy, the operation time of the cough group was longer. (**B**) In the lobectomy group, the cough group had more blood loss. (**C**) Among the pulmonary resection subgroups, there was no difference in the chest drainage duration and hospital stay between the cough group and the non-cough group (**D**).

The patients were further analyzed according to type of pulmonary resection (Figure 1). Among the patients who received wedge resection, those with and without postoperative cough were not significantly different in terms of operation time (*p* = 0.540), blood loss (*p* = 0.393), postoperative drainage time (*p* = 0.053), or duration of hospital stay (*p* = 0.767) (Figure 1). Among the patients who underwent segmentectomy, those with and those without postoperative cough were not significantly different in terms of operation time (*p* = 0.312), blood loss (*p* = 0.783), postoperative drainage time (*p* = 0.107), or duration of hospital stay (*p* = 0.073). However, among patients who underwent lobectomy, those with postoperative cough had a longer operation time (*p* = 0.009) and more blood loss (*p* = 0.034) than the non-cough group, but those with and without postoperative cough were not significantly different in terms of drainage time (*p* = 0.838) or duration of hospital stay (*p* = 0.826).

Furthermore, as shown by the univariate analysis of cough in the patients who underwent lobectomy (Table 3), the patients with postoperative cough and the patients without postoperative cough were comparable in terms of sex, age, smoking status, drinking status, BMI, FEV1, FEV1%, CCI, tumor size, tumor location, lymph node metastasis, stage 7 lymph node metastasis, postoperative drainage time, and hospitalization time. The patients with postoperative cough had a higher rate of systematic lymph node dissection than the patients without postoperative cough (86.43% vs. 38.56%. χ^2^ = 70.697, *p* < 0.001). The patients with postoperative cough had more blood loss (158.96 ± 117.34 mL vs. 135.59 ± 64.94 mL; *p* = 0.034) and longer operation time (156.31 ± 40.01 min vs. 143.78 ± 41.23 min; *p* = 0.009).

Furthermore, as shown by the univariate analysis of cough in the patients with NSCLC (Table 4), the patients with and without postoperative cough were comparable in terms of sex, age, smoking status, drinking status, BMI, tumor size, tumor location, lymph node metastasis, and stage 7 lymph node metastasis. Compared to the patients without postoperative cough, those with postoperative cough had a higher incidence of lobectomy (64.38% vs. 46.26%, χ^2^ = 12.23, *p* = 0.006), a higher incidence of lymph node dissection (67.50% vs. 35.37%, χ^2^ = 31.685, *p* < 0.001), more blood loss (150.66 ± 107.24 mL vs. 133.81 ± 66.09 mL; *p* = 0.018), and longer operation time (150.90 ± 39.49 min vs. 138.98 ± 40.79 min; *p* = 0.010).

### 3.3. Analysis of the Short-Term Postoperative LCQ Score

**Figure 2:**
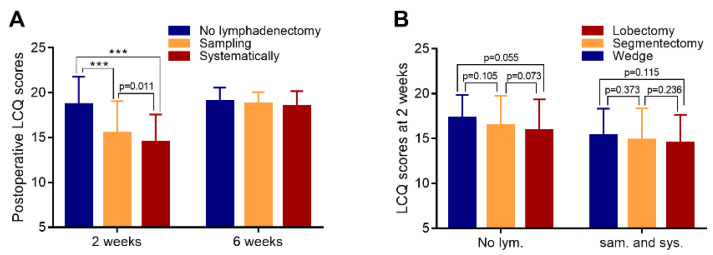
LCQ scores during follow-up. (**A**) LCQ scores of each subgroup at 2 and 6 weeks after surgery (subgroups were classified according to the lymph node management method). (**B**) LCQ scores at 2 weeks after surgery (subgroups were classified according to whether lymph node resection was performed). *** denote *p* < 0.001.

The LCQ scores of the patients at 2 weeks after surgery were analyzed (Figure 2A). The mean preoperative LCQ score of patients who did not undergo lymph node resection was higher than that of the patients who underwent lymph node sampling (18.69 ± 3.10 vs. 15.47 ± 3.58; *p* < 0.001) and systematic lymph node dissection (18.69 ± 3.10 vs. 14.49 ± 3.09; *p* < 0.001). The mean LCQ of the patients who underwent lymph node sampling was also higher than that of the patients who underwent systematic lymph node dissection (15.47 ± 3.58 vs. 14.49 ± 3.09; *p* = 0.011). At 6 weeks after surgery (Figure 2A), there was no difference in LCQ scores between patients treated using different lymph node management methods.

Among the patients who did not undergo lymph node resection (Figure 2B), there was no difference in LCQ scores between those treated with different surgical methods (*p* > 0.05). Similarly, among the patients who underwent lymph node resection (including lymph node sampling and systematic lymph node resection), there was no difference in LCQ scores between patients treated with different surgical methods (*p* > 0.05).

### 3.4. Multivariate Regression Analysis of Cough after Pulmonary Resection and Changes of Postoperative Cough State

A multivariate logistic regression analysis of the risk factors for postoperative cough was performed. The results showed that the lymph node resection method is a risk factor for postoperative cough (odds ratio 3.677, 95% confidence interval: 2.514–5.378, *p* < 0.001) (Table 5).

**Figure 3:**
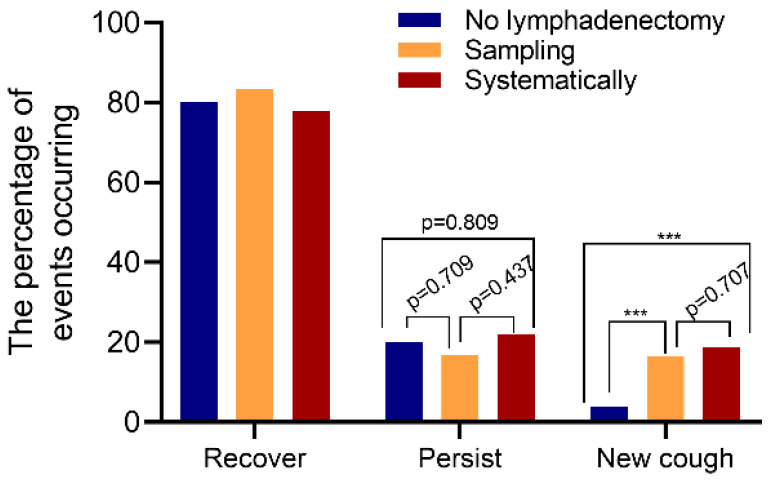
Statistical analysis of the incidence of cough events between groups. There were no differences in the recovery rate of postoperative cough (Left three columns) and no differences in the persist rate of postoperative cough (Middle three columns). In patients with no postoperative cough, the recurrence rate of cough in the no lymphadenectomy group was significantly lower than that in the sampling group and systematic lymph node dissection group (Right three columns). *** denote *p* < 0.001.

Furthermore, we explored the changes of postoperative cough status (Table 6). We found no differences in the recovery rate of postoperative cough or in the persistence of cough among the different lymph node treatment groups. However, in patients with no postoperative cough, the recurrence rate of cough in the no lymphadenectomy group was significantly lower than in the sampling group (3.9% vs. 16.4%, *p* < 0.001) and systematic lymph node dissection group (3.9% vs. 18.8%, *p* < 0.001), respectively (Figure 3). There was no difference between the sampling and systematic lymph node dissection group.

## 4. Discussion

To our knowledge, this study is a large-sample study of the risk factors for cough after pulmonary resection by VATS. Our study showed that the lymph node resection method is an independent risk factor for short-term postoperative cough, while gender, age, smoking and drinking history, pulmonary resection method, operation time, blood loss, and lymph node metastasis are not. Furthermore, patients in different groups effectively recovered from cough 6 weeks after surgery.

Cough is a common complication after pulmonary resection [21]. To exclude the effect of preoperative cough symptoms, we excluded patients with preoperative cough to better investigate postoperative cough caused by pulmonary resection. Some studies have shown that compared with males, females are more likely to develop preoperative cough that persists longer [5] because of hormonal influences and high visceral sensitivity, along with a hypersensitivity of airway afferents to the somatosensory cortex [22,23]; as a result, the quality of life of female patients is more negatively impacted by preoperative cough. Our study showed that sex was not a factor associated with postoperative cough, which is consistent with the results of Xie et al. [10]. There also was no significant difference about postoperative cough between females and males after thyroidectomy [24]. This may be related to the bias in patient inclusion. In our study, we included 365 males and 152 females which represented a relatively large sample size. It may have a higher reliability. Further meta-analysis or prospective controlled studies focusing only on the relationship between gender and cough may be necessary.

Our study showed that different tumor locations (which correspond largely to different excision extensions) did not have different effects on postoperative cough. Damage to the blood vessels and nerves of the anterior tracheal wall as a result of dissection of the upper mediastinal lymph nodes has been reported to be a factor that causes postoperative chronic cough after right upper lung lobectomy [10]. However, among the patients in our study who underwent systematic lymph node dissection, the same lymph node dissection procedure was applied to the right upper lung, right lower lung, and right middle lobe. Since the extent of lymph node dissection was the same, there should be no difference in the mediastinal nerve damage caused by lymph node dissection. Therefore, we believe that different pulmonary lobe resection has no differential effects on postoperative cough.

Our univariate analysis results showed that a longer operation time, a higher blood loss volume, and malignancy tumor are all factors influencing postoperative cough, which is consistent with the results of Chen et al. [11,25]. During anesthesia, stimulation of the trachea by tracheal intubation or extubation, opioid use [26] and the toxic effects of inhaled anesthetics [27] are considered possible causes of postoperative cough. Pulmonary lobectomy combined with systematic lymph node dissection generally takes longer to perform than wedge resection or lymph node sampling and is therefore associated with prolonged anesthesia times. In addition, compared with benign diseases, malignant tumors require more extensive resection and dissection of more lymph nodes, which leads to prolonged drainage time and hospital stays [28]. Therefore, we believe that more aggressive surgical methods (lobectomy) and a wider resection range (systematic lymph node dissection) are factors that influence postoperative cough. We further analyzed the risk factors for postoperative cough in the pulmonary lobectomy subgroup and in the malignant tumor subgroup. The findings were consistent with the previous results. However, postoperative cough was not related to the resected lobe (tumor location), lymph node metastasis, or stage 7 lymph node metastasis.

The multivariate analysis of factors that may affect postoperative cough showed that lymph node dissection is an independent risk factor. A study by Sawabata et al. showed that mediastinal lymph node resection may contribute to coughing after pulmonary resection, mainly because of damage to the vagus nerve and its branches during lymph node resection [29]. Cough receptors may be mainly located in the larynx, trachea, carina, and large pulmonary bronchi [30]. During lymph node dissection, damage to the vagus nerve fibers or receptors which disrupts the neural reflex pathways of cough results in postoperative cough. This is consistent with the results of our study.

We further used the LCQ to describe postoperative cough symptoms [31] and observe them during follow-up. At 6 weeks after surgery, the cough symptoms were largely alleviated. Further subgroup analysis of different lymph node management methods showed that when the same lymph node management method was used, different surgical methods did not have a significantly different effect on postoperative cough. Interestingly, delayed cough was more likely to occur when lymph node dissection was performed (in both the sampling and systematic groups), even if there was no postoperative cough. To our knowledge, this has not been reported in previous studies. This further confirms that lymph node resection is the most important risk factor for postoperative cough after pulmonary resection. Delayed cough after pulmonary resection may be a focus of our future research.

Lymph node dissection is a very important procedure in the operation of NSCLC [32,33]. The pulmonary branches of the vagus nerve are mainly divided into the anterior plexus and the posterior plexus [34], which enter the lungs through the mediastinum along the trachea and bronchi. Lymph node resection may cause damage to the vagus nerve or its branches, thereby causing postoperative cough. However, when pulmonary resection was performed without lymph node resection, vagus nerve injury rarely occurred, and the postoperative cough symptoms were milder.

According to the location and distribution of the vagus nerve, mediastinal lymph node dissection is usually the main cause of its injury during pulmonary resection. Clinically, we found that cough in patients after pulmonary resection was related to surgical trauma, especially the injury to the vagus nerve and its pulmonary branches, rather than the trauma of lung surgery itself and the location or the number of incisions. These two groups were also analyzed separately in this study. In addition, some researchers have reported whether the use of intraoperative energy devices causes different nerve injury. Andreas Manouras et al. [35] conducted a comparative analysis between the electrothermal bipolar vessel sealing system, harmonic scalpel and classic suture ligation showed that there was no significant difference between the three methods in the treatment of superior and inferior laryngeal nerves, so all of them were safe and feasible. However, Arulalan Mathialagan et al. [36] reported that in patients undergoing selective neck dissection for primary oral malignancy, nerve injury was less, and spinal accessory nerve function recovery was better in harmonic scalpel group as compared to the electro cautery group. Intraoperative nerve monitoring (IONM) has been applied in many operations, such as thyroid surgery [37,38], esophageal cancer surgery for recurrent laryngeal nerve protection [39,40], and craniocerebral surgery [41]; however, it is rarely used in pulmonary surgery. The reason might be that cough in patients after surgery had a good long-term recovery, while researchers ignored cough that may have troubled patients in the short term, or even persistent long-term cough of some patients. In future studies, we hope to conduct a comparative analysis of different intraoperative energy devices, and to use IONM to make more accurate classification of nerve injury in the research process and to protect the vagus nerve more accurately.

These findings prompted our thoracic surgeons to reflect on whether avoiding damage to the vagus nerve and its branches during surgery, especially during treatment of the hilar pleura, could reduce the occurrence of postoperative cough, especially persistent, severe cough symptoms. The most important means of avoiding such damages would be to expose the anatomy of the vagus nerve as clearly as possible and to preserve as many vagus nerve-innervated pulmonary branches as possible because clear exposure of the anatomy is the best way to ensure the safety of operations [42].

Our study has some limitations. First, we did not evaluate preoperative cough but only included patients who did not complain of cough symptoms. Second, we did not evaluate reflux in the patients. Third, we did not conduct a follow-up evaluation of long-term cough symptoms. In future studies, we will improve these limitations.

## 5. Conclusions

Lymph node resection is an independent risk factor for short-term cough after pulmonary resection by VATS, possibly due to damage to the vagus nerve and its branches (particularly the pulmonary branches). The mechanisms and characteristics of cough after pulmonary resection require further clarification.

## Figures and Tables

**Table 1:** Clinical characteristics and univariate analysis in patients with or without postoperative cough.

Characteristics	Total (%)	No Cough (%)	Cough (%)	χ^2^/t	*p*-Value
Patients (*n*)	517	310 (59.96)	207 (40.04)		
Gender (*n* (%))				1.826	0.177
Male	365 (70.60)	212 (68.39)	153 (73.91)		
Female	152 (29.40)	98 (31.61)	54 (26.09)		
Age (years)		59.18 ± 9.50	57.81 ± 9.59	1.601	0.110
Smoking status				0.008	0.930
Absent	226 (43.71)	136 (43.87)	90 (43.48)		
Present	291 (56.29)	174 (56.13)	117 (56.52)		
Drinking status				0.876	0.349
Absent	200 (38.68)	125 (40.32)	75 (36.23)		
Present	317 (61.32)	185 (59.68)	132 (63.77)		
BMI (kg/m^2^)		22.45 ± 3.12	22.41 ± 2.86	0.133	0.894
FEV1 (L)		2.04 ± 0.43	1.95 ± 0.41	1.065	0.288
FEV1 %		85.42 ± 15.74	83.68 ± 14.60	1.235	0.218
CCI		2.02 ± 1.34	1.95 ± 1.45	0.609	0.543
Tumor size (cm)		2.03 ± 0.91	2.44 ± 3.79	−1.835	0.067
Tumor location					
Right upper lobe	166 (32.11)	100 (32.26)	66 (31.88)	2.538	0.638
Right middle lobe	26 (5.03)	16 (5.16)	10 (4.83)		
Right lower lobe	107 (20.70)	66 (21.29)	41 (19.81)		
Left upper lobe	122 (23.60)	77 (24.84)	45 (21.74)		
Left lower lobe	96 (18357)	51 (16.45)	45 (21.74)		

Abbreviations: BMI, body mass index; FEV1, forced expiratory volume in 1 s; CCI, Charlson comorbidity index.

**Table 2:** Comparison of the treatment results between the two groups.

Results	No Cough (*n* = 310)	Cough (*n* = 207)	χ^2^/τ	*p*-Value
Operation time (min)	122.03 ± 53.55	146.11 ± 46.72	−5.412	0.000
Blood loss (mL)	113.85 ± 71.47	142.46 ± 106.01	−3.666	0.000
Tumor			45.929	0.000
Benign	163 (52.58)	47 (22.71)		
Malignant	147 (47.42)	160 (77.29)		
Type of incision			1.545	0.214
Single-port	173 (55.81)	104 (50.24)		
Two-port	137 (44.19)	103 (49.76)		
Type of resection			23.594	0.000
Wedge resection	68 (21.94)	13 (6.28)		
Segmentectomy	89 (28.71)	54 (26.09)		
Lobectomy	153 (49.35)	140 (67.63)		
Type of lymphadenectomy			118.04	0.000
No lymphadenectomy	179 (57.74)	30 (14.49)		
Sampling	71 (22.90)	50 (24.15)		
Systematically	60 (19.36)	127 (61.36)		
Drainage (days)	3.87 ± 2.33	4.34 ± 2.96	−2.202	0.028
Hospital stay (days)	7.58 ± 3.84	8.32 ± 3.81	−2.121	0.034

**Table 3:** Univariate analysis in patients with lobectomy.

Characteristics	No Cough (*n* = 153)	Cough (*n* = 140)	χ^2^/τ	*p*-Value
Gender (*n* (%))			0.289	0.591
Male	106 (69.28)	101 (72.14)		
Female	47 (30.72)	39 (27.86)		
Age (years)	59.54 ± 8.92	57.56 ± 9.33	1.996	0.057
Smoking status			0.028	0.867
Absent	63 (41.18)	59 (42.14)		
Present	90 (58.82)	81 (57.86)		
Drinking status			0.382	0.536
Absent	60 (39.22)	50 (35.71)		
Present	93 (90.78)	90 (64.29)		
BMI (kg/m^2^)	22.26 ± 3.04	22.66 ± 2.80	−1.149	0.252
FEV1 (L)	2.05 ± 0.49	1.99 ± 0.43	1.135	0.257
FEV1 %	86.58 ± 15.52	83.95 ± 15.96	1.428	0.154
CCI	2.08 ± 1.33	1.92 ± 1.49	0.953	0.341
Tumor size (cm)	2.60 ± 0.87	2.93 ± 4.52	−0.89	0.374
Tumor location				
Right upper lobe	53 (34.64)	44 (31.43)	3.003	0.557
Right middle lobe	9 (5.88)	6 (4.29)		
Right lower lobe	36 (23.53)	30 (21.43)		
Left upper lobe	28 (18.30)	24 (17.14)		
Left lower lobe	27 (17.65)	36 (25.71)		
Type of lymphadenectomy			70.697	0.000
No lymphadenectomy	94 (61.44)	19 (13.57)		
Systematically	59 (38.56)	121 (86.43)		
lymphatic metastasis			1.994	0.158
Negative	50 (73.53)	85 (82.52)		
Positive	18 (26.47)	18 (17.48)		
Stage 7 lymph node metastasis			0.839	0.360
Negative	51 (86.44)	110 (90.91)		
Positive	8 (13.56)	11 (9.09)		
Operation time (min)	143.78 ± 41.23	156.31 ± 40.01	−2.636	0.009
Blood loss (mL)	135.59 ± 64.94	158.96 ± 117.34	−2.133	0.034
Drainage (days)	4.31 ± 2.83	4.38 ± 3.13	−0.205	0.838
Hospital stay (days)	8.41 ± 4.40	8.31 ± 3.51	0.22	0.826

Abbreviations: BMI, body mass index; FEV1, forced expiratory volume in 1 s; CCI, Charlson comorbidity index.

**Table 4:** Univariate analysis in patients with NSCLC.

Characteristics	No Cough (*n* = 147)	Cough (*n* = 160)	χ^2^/τ	*p*-Value
Gender (*n* (%))			2.205	0.138
Male	96 (65.31)	117 (73.13)		
Female	51 (34.69)	43 (26.88)		
Age (years)	59.36 ± 10.48	58.44 ± 9.96	0.791	0.429
BMI (kg/m^2^)	22.22 ± 3.10	22.40 ± 2.73	−0.545	0.586
Tumor size (cm)	2.04 ± 0.98	2.42 ± 4.29	−1.061	0.290
Tumor location				
Right upper lobe	46 (31.29)	47 (29.38)	2.462	0.652
Right middle lobe	6 (4.08)	7 (4.38)		
Right lower lobe	36 (24.49)	39 (24.38)		
Left upper lobe	37 (25.17)	33 (20.63)		
Left lower lobe	22 (14.97)	34 (21.23)		
Type of resection			12.23	0.006
Wedge resection	10 (6.80)	3 (1.88)		
Segmentectomy	69 (46.94)	54 (33.75)		
Lobectomy	68 (46.26)	103 (64.38)		
Type of lymphadenectomy			31.685	0.000
Sampling	95 (64.63)	52 (32.50)		
Systematically	52 (35.37)	108 (67.50)		
lymphatic metastasis			0.395	0.530
Negative	125 (85.03)	140 (87.50)		
Positive	22 (14.97)	20 (12.50)		
Stage 7 lymph node metastasis			1.748	0.253
Negative	115 (93.50)	153 (96.84)		
Positive	8 (6.50)	5 (3.16)		
Operation time (min)	138.98 ± 40.79	150.90 ± 39.49	−2.601	0.010
Blood loss (mL)	133.81 ± 66.09	150.66 ± 107.24	−1.640	0.018

Abbreviations: BMI, body mass index; FEV1, forced expiratory volume in 1 s; CCI, Charlson comorbidity index.

**Table 5:** Multivariate logistic regression analysis in patients with or without postoperative cough.

Variables *	B	S.E.	Wals	OR (95% CI)	*p*-Value
Tumor size	0.009	0.043	0.440	1.009 (0.927–1.099)	0.835
Operation time	0.003	0.003	1.612	1.003 (0.998–1.009)	0.204
Blood loss	0.000	0.001	0.123	1.000 (0.997–1.002)	0.726
Pathologic types	−0.032	0.303	0.011	0.969 (0.535–1.755)	0.916
Type of resection	−0.204	0.212	0.931	0.815 (0.538–1.235)	0.335
Type of lymphadenectomy	1.302	0.194	45.079	3.677 (2.514–5.378)	0.000
Drainage time	0.058	0.048	1.464	1.060 (0.965–1.164)	0.226
Hospital stay	0.021	0.033	0.413	1.022 (0.957–1.091)	0.521

* Logistic regression test, variables whose *p*-value less than 0.1 were included.

**Table 6:** Changes of postoperative cough state from 3 days to 6 weeks after surgery.

Groups	Cases (*n*)	3 POD	6 Weeks	Cough Recover	Cough Persist	Develop a New Cough
No lymphadenectomy	209					
No cough		179	196			7 (3.9%)
Cough		30	13	24 (80.0%)	6 (20.0%)	
Sampling	121					
No cough		73	101			12 (16.4%)
Cough		48	20	40 (83.3%)	8 (16.7%)	
Systematic	187					
No cough		69	148			13 (18.8%)
Cough		118	39	92 (78.0%)	26 (22.0%)	

## Data Availability

Data is contained within the article.
